# Signal mining and analysis of trifluridine/tipiracil adverse events based on real-world data from the FAERS database

**DOI:** 10.3389/fphar.2024.1399998

**Published:** 2024-07-23

**Authors:** Yongli Hu, Yan Du, Zhisheng Qiu, Chenglou Zhu, Junhong Wang, Tong Liang, Tianxiang Liu, Mingxu Da

**Affiliations:** ^1^ The First Clinical Medical College of Lanzhou University, Lanzhou University, Lanzhou, China; ^2^ Department of Gastrointestinal Surgery, Affiliated Hospital of Guilin Medical University, Guilin, China; ^3^ The Second Clinical Medical College of Lanzhou University, Lanzhou University, Lanzhou, China; ^4^ Department of Oncology Surgery, Gansu Provincial Hospital, Lanzhou, China

**Keywords:** FAERS database, trifluridine/tipiracil, adverse events, disproportionality analysis, pharmacovigilance

## Abstract

**Objective:**

The objective of this research is to scrutinize adverse events (AEs) linked to Trifluridine/Tipiracil (TFTD/TPI), using data from the FDA Adverse Event Reporting System (FAERS) database.

**Methods:**

The AEs data related to TFTD/TPI were collected from the fourth quarter of 2015 through the fourth quarter of 2023. After normalizing the data, multiple signal quantification techniques including Proportional Reporting Ratio (PRR), Reporting Odds Ratio (ROR), Bayesian approaches such as Bayesian Confidence Propagation Neural Network (BCPNN) and the Multi-item Gamma Poisson Shrinker (MGPS) were used for overall and subgroup analysis and visualization analyses were performed.

**Results:**

From the FAERS database, we analyzed 13,520,073 reports, identifying 8,331 as primary suspect (PS) AEs for TFTD/TPI, occurring across 27 organ systems. The study retained 99 significant disproportionality Preferred Terms (PTs) across four algorithms and unveiled unexpected serious AEs such as iron deficiency and intestinal perforation, hepatic failure, cholangitis and so on. The median onset of TFTD/TPI-associated AEs was 44 days (IQR 20-97 days), with most occurring within the first 30 days of treatment.

**Conclusion:**

This research uncovers critical new safety signals for TFTD/TPI, supporting its clinical monitoring and risk identification.

## 1 Introduction

According to the latest data released by the WHO, the number of new cancer cases worldwide in 2022 will reach 20 million, with 9.7 million deaths. Among them, the incidence of colorectal cancer will be 1.9 million and the number of deaths will exceed 900,000, with the new incidence rate and mortality rate occupying the third and second places respectively (World Health Organization, 2024). Initially, 20% of colorectal cancer patients are diagnosed with metastatic disease, with a further 50% developing metastasis from locally advanced cancer (Ciardiello et al., 2022). The 5-year survival rate for metastatic colorectal cancer is under 20% (Biller and Schrag, 2021), highlighting the importance of non-surgical treatment strategies. Treatment varies by metastasis location and patient health, involving chemotherapy, radiation, surgery, and newer therapies like targeted and immunotherapy, aiming for a comprehensive approach to improve outcomes.

Advancements in sequencing technologies have refined metastatic colorectal cancer classification, supporting pharmacotherapy development. This progress has notably enhanced patient survival rates (Cann et al., 2023). Chemotherapy combinations, including fluorouracil, are fundamental in treating metastatic colorectal cancer (Glimelius et al., 2021). The long duration of treatment, significant tumor load, and genetic mutations can lead to fluorouracil resistance, a key challenge in managing advanced stages of the disease.

TAS102, or Trifluridine/Tipiracil (FTD/TPI), is a chemotherapy drug for colorectal cancer, akin to oral fluorouracil medications like capecitabine and S-1. It uniquely combines trifluridine, which inhibits tumor cell DNA replication, and tipiracil hydrochloride, enhancing trifluridine’s bioavailability by preventing its degradation. This combination effectively targets and kills tumor cells (Chen et al., 2016). A global Phase III clinical trial involving 800 patients with refractory metastatic colorectal cancer experienced significantly better disease control rates (44% vs. 16%) and longer survival (7.1 months vs. 5.3 months) with FTD/TPI compared to placebo, reducing the risk of death by 32% (Mayer et al., 2015). Based on the results of this study, Trifluridine/tipiracil was FDA approved in 2015 for metastatic colorectal cancer (mCRC) patients previously treated with fluoropyrimidine-, oxaliplatin-, and irinotecan-based chemotherapy, and for those who have had or are unsuitable for anti-VEGF and anti-EGFR treatments (in RAS wild-type cases) (Bachet et al., 2020). Another Phase III clinical trial, exclusively involving Asian populations, indicated that the FTD/TPI group had a significantly lower risk of death compared to the placebo group (HR = 0.79, 95% CI: 0.62–0.99, *p* = 0.035), with a median overall survival of 7.8 months for FTD/TPI vs. 7.1 months for placebo. Pre-specified subgroup analysis of OS suggested a favorable trend in chemotherapy (FTD/TPI) effectiveness across all parameters except age, with similar rates of serious adverse events between both groups (Xu et al., 2018).

Although FTD/TPI has been widely used in fluoropyrimidine-resistant refractory colorectal cancer, reports of adverse events (AEs) have increased. The common adverse reactions and abnormal laboratory findings include anemia, neutropenia, fatigue, nausea, thrombocytopenia, decreased appetite, diarrhea, vomiting, abdominal pain, and fever (Mayer et al., 2015; Xu et al., 2018; Kröning et al., 2023). Studies have shown that Grade ≥3 neutropenia, after adjusting for age and the modified Glasgow prognostic score, is associated with longer survival periods (Watanabe et al., 2023). Anemia and neutropenia are more common among patients with renal impairment (Van Cutsem et al., 2022).

While there have been case reports, clinical trials, and meta-analyses on the efficacy and safety of FTD/TPI (Mohamed, 2023, Van Cutsem et al., 2022; Shitara et al., 2024), these studies were conducted under specific systems, with relatively limited sample sizes and specific inclusion criteria. Comprehensive safety data from large samples or real-world cohorts are currently lacking. Hence, employing data mining algorithms for pharmacovigilance analysis to assess the safety of FTD/TPI in real-world settings has become necessary.

The FDA Adverse Event Reporting System (FAERS) is a database that collects adverse event and medication error reports submitted to the FDA (Fusaroli et al., 2024). It is a tool for the FDA to monitor and improve the safety of drugs and therapeutic biological products. The data in FAERS support the FDA’s post-marketing safety surveillance program for drug and therapeutic biologic products, allowing for the identification of new safety information or trends. Healthcare professionals and consumers can report adverse events, which are then included in the database to aid in the evaluation of the risks and benefits of these products. The aim of this study is to evaluate and compare the safety profile of FTD/TPI by analyzing adverse events reported in the FAERS database related to FTD/TPI use.

## 2 Materials and methods

### 2.1 Data sources

Data for pharmacovigilance research on FTD/TPI was downloaded and obtained from the FAERS database (https://www.fda.gov/drugs/questions-and-answers-fdas-adverse-event-reporting-system-faers) covering the period from the fourth quarter of 2015 to the fourth quarter of 2023, focusing on the post-marketing environment of FTD/TPI.

**FIGURE 1:**
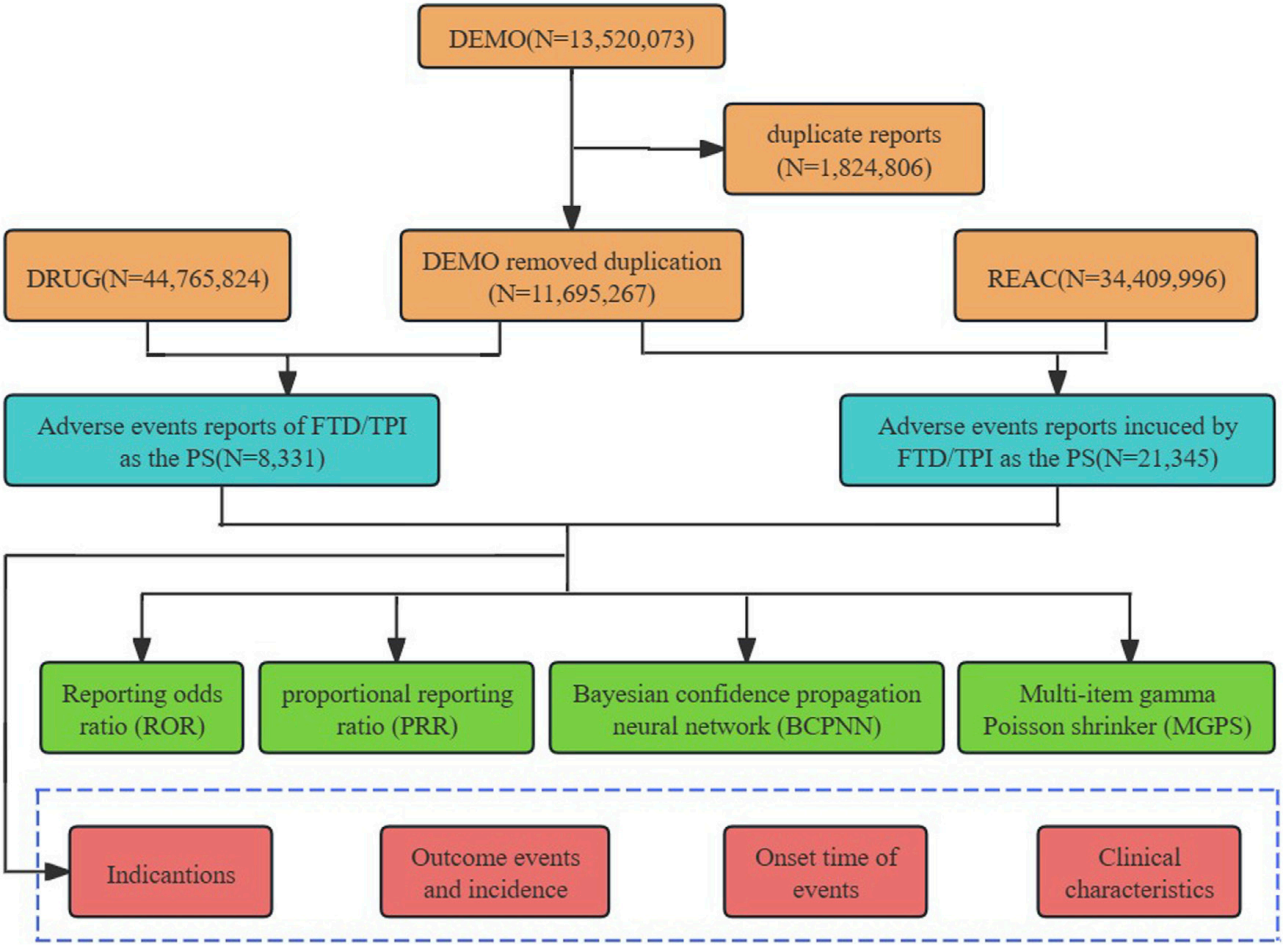
Process for selecting TFTD/TPI related adverse events (AEs) from the FAERS database.

The FAERS database aligns with the ICH’s International Safety Reporting Guidelines (ICH E2B), utilizing the MedDRA for coding adverse events. It comprises seven distinct data files: demographic and administrative information (DEMO), drug information (DRUG), adverse event coding (REAC), patient outcomes (OUTC), report sources (RPSR), therapy start and end dates for reported drugs (THER), and indications for drug administration (INDI). To eliminate duplicates, we prioritize reports based on the most recent FDA_DT per CASEID, or opt for the highest PRIMARYID in cases of identical CASEID and FDA_DT dates, ensuring data integrity and reliability (Zou et al., 2023). In the pharmaceutical documentation, the generic name (Trifluridine/Tipiracil), abbreviation (TAS102, FTD/TPI), and trade name (Lonsurf) are defined as the target search terms for the drug. Drugs identified in reports are categorized into primary suspect (PS), secondary suspect (SS), concomitant (C), and interacting (I) based on their relation to the event. During the study period, totally 13,520,073 reports of FTD/TPI were gained from FAERS database. 374,322 case reports of FTD/TPI as the primary suspect (PS) drug after the exclusion of duplicates, and 8,331 AEs were associated with FTD/TPI. AEs reported involving FTD/TPI were classified at both the system organ class (SOC) and preferred term (PT) levels based on the Medical Dictionary for Regulatory Activities (MedDRA, version 26.0). A comprehensive analysis of 21,345 terms associated with FTD/TPI identified them as preferred terms (PTs). The overall analysis process is shown in Figure 1.

### 2.2 Data analytics

Pharmacovigilance relies on disproportionality analysis, using methods like Proportional Reporting Ratio (PRR) and Reporting Odds Ratio (ROR) for signal detection, alongside Bayesian approaches such as Bayesian Confidence Propagation Neural Network (BCPNN) and the Multi-item Gamma Poisson Shrinker (MGPS) for a more robust analysis. PRR and ROR offer simplicity and are suited for small samples, respectively, but may face bias or complexity in calculations. BCPNN and MGPS provide a statistical foundation and handle sparse data efficiently, enhancing true signal detection. Combining these algorithms allows for a comprehensive evaluation of drug safety, with precise detection parameters outlined for clarity. This strategic approach facilitates the identification of reliable safety signals from the data. Four-compartment table of drugs and adverse reactions, utilized for evaluating the relationship between a particular medication and the incidence of a specific adverse event (Supplementary Table S1). For clarity and accuracy, the exact formulas and criteria used in this study were displayed in Supplementary Table S2.

### 2.3 Time to onset analysis

The time to onset (TTO) of FTD/TPI induced AEs was defined as the time between START_DT (the date of FTD/TPI initiation in the THER file) and EVENT_DT (the date of onset of AEs in the DEMO file). Removed data inaccurate or missing dates entered and EVENT_DT earlier than START_DT.

Data were processed and visualized using R software (version 4.2.1) Microsoft EXCEL 2010, GraphPad Prism 8, “ggplot2”package.

## 3 Results

### 3.1 General features

Clinical characteristics of FTD/TPI-associated AEs are listed in Table 1.

**TABLE 1:** Clinical characteristics of reports with Trifluridine/Tipiracil from the FAERS database.

Characteristics	Case number, n	Case proportion (%)
Overall	8331	
Sex		
Female	3503	42.00
Male	4753	57.10
Missing	75	0.90
Age		
<18	1	0.01
18 ≥ and < 65	3074	36.90
≥65	3138	37.67
Missing	2118	25.42
Outcome		
Death	3501	42.02
Life-Threatening	52	0.62
Disability	13	0.16
Hospitalization - Initial or Prolonged	1814	21.77
Other Serious	849	10.19
Missing	2102	25.23
Indications (TOP seven)*		
Colon cancer	3469	31.40
Products for unknown indications	1255	11.36
Rectal cancer	1042	9.43
Colorectal cancer metastatic	1001	9.06
Colon cancer metastatic	312	2.82
Colorectal cancer	285	2.58
Rectosigmoid cancer	213	1.93
Reported Person		
Consumer	3541	42.50
Other health-professional	1576	18.90
Pharmacist	1542	18.50
Physician	1170	14.00
Health Professional	454	5.40
Lawyer	1	0.01
Missing	47	0.60
Reported Countries(Top five)		
America	6834	82.00
Canada	627	7.50
Japan	208	2.50
France	99	1.20
Denmark	90	1.10

**FIGURE 2:**
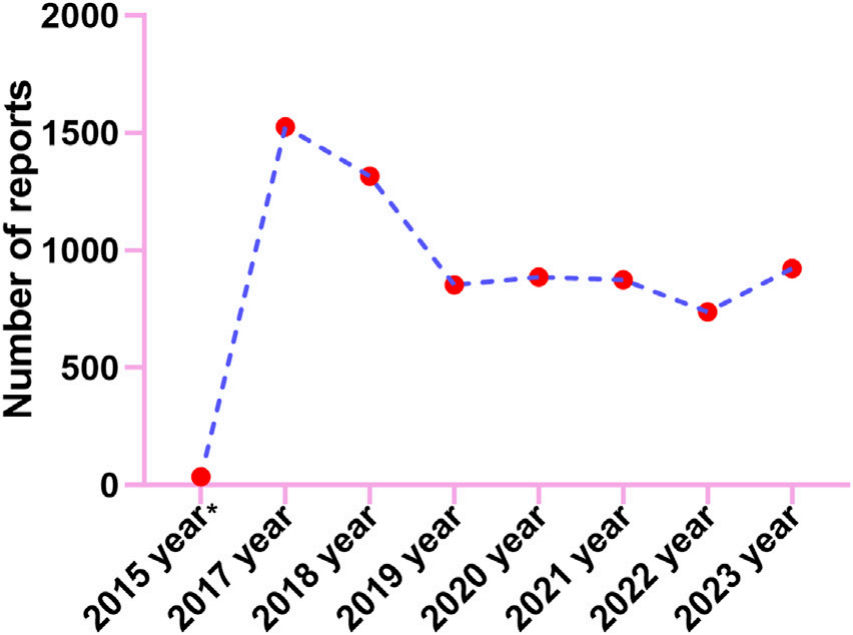
The annual distribution of TFTD/TPI related adverse events reported from 2015 (Q4) through 2023.

Adverse events (AE) occurrence among males (57.10%) surpassed those in females (42.00%), indicating a higher prevalence of AEs in males patients. Besides, the report describes a slightly higher proportion of AEs seen the elderly patients (37.67% of patients >65 years old) compared to 18≥ and <65 years old (36.98%). We calculate the ratio of each specific serious outcome to the total number of serious outcome reports to ascertain its prevalence. The most common serious outcome was death, accounting for 42.02% of reports, likely linked to disease advancement from the tumor. Reports of hospitalizations and other serious outcomes constituted 21.77% and 10.19% respectively (Supplementary Figure S1A). From reported indications, the top ones were all related to colon and rectal cancer and their metastatic tumors (Supplementary Figure S1B). Consumers reported the largest number of adverse events at 42.50% (Supplementary Figure S1C). Geographically, America reported the highest percentage of 82.00%, followed by Canada, Japan, France, and Denmark with 7.50%, 2.50%, 1.20%, and 1.10% respectively (Supplementary Figure S1D). The reported year data showed a peak in case numbers, reaching 1526 in 2017, followed by a decline started in 2018, with figures then leveling off (Figure 2).

### 3.2 System organ class signals

**FIGURE 3:**
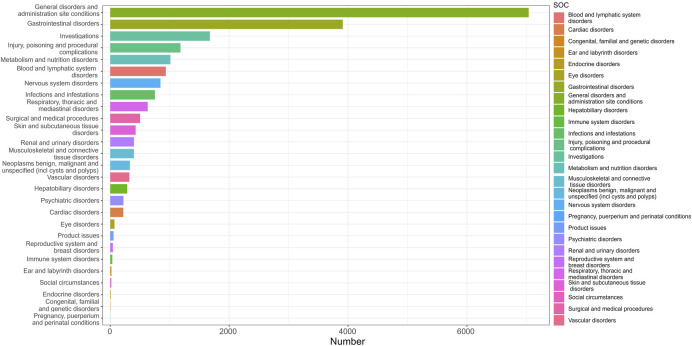
The distribution of System Organ Classes (SOCs) quantity.

The analysis revealed that AEs resulted from FTD/TPI usage impacted 27 System Organ Classes (SOCs). Figure 3 displays the frequency of adverse events (AEs) by SOCs, succinctly showing their occurrence across different organ systems. Table 2 presents the signal intensities of FTD/TPI associated AEs across different SOC. Within this study, various SOCs were deemed significant based on their fulfillment of one or more criteria from the four indices utilized for the analysis. Several vital SOCs included: General disorders and administration site conditions (N = 7038, ROR [2.2, 95% CI 2.20-2.33]), Gastrointestinal disorders (N = 3911, ROR [2.49, 95% CI 2.41-2.58]), Investigations (N = 1676, ROR [1.38, 95% CI 1.31-1.45]), Metabolism and nutrition disorders (N = 1012, ROR [2.41, 95% CI 2.27-2.57]), Blood and lymphatic system disorders (N = 935, ROR [2.76, 95% CI 2.59-2.95]), Surgical and medical procedures (N = 502, ROR [1.72, 95% CI 1.57-1.87]), Hepatobiliary disorders (N = 286, ROR [1.66, 95% CI 1.48-1.87]). The analysis underscores the primary organ systems affected by FTD/TPI related AEs, pinpointing the need for enhanced scrutiny and research in these areas to better understand and mitigate these effects.

**TABLE 2:** Signal strength of reports of Trifluridine/Tipiracil at the System Organ Class (SOC) level in FAERS database.

System organ class (SOC) name	Cases reporting SOC	ROR (95% two-sided CI)	PRR (χ2)	EBGM (EBGM 05)	IC(95%CI lower limit)
General disorders and administration site conditions	7038	2.27 (2.20–2.33)*	1.85 (3336.51)	1.85 (1.80)	0.89 (-0.78)
Gastrointestinal disorders	3911	2.49 (2.41–2.58)*	2.22 (2849.52)*	2.22 (2.14)*	1.15 (-0.52)
Investigations	1676	1.38 (1.31-1.45)*	1.35 (163.10)	1.35 (1.29)	0.44 (-1.23)
Injury, poisoning and procedural complications	1181	0.44 (0.42–0.47)	0.47 (781.85)	0.47 (0.45)	−1.08 (-2.74)
Metabolism and nutrition disorders	1012	2.41 (2.27–2.57)*	2.35 (797.71)*	2.35 (2.20)*	1.23 (-0.44)
Blood and lymphatic system disorders	935	2.76 (2.59–2.95)*	2.68 (1002.71)*	2.68 (2.51)*	1.42 (-0.24)
Nervous system disorders	845	0.49 (0.46–0.52)	0.51 (433.54)	0.51 (0.48)	−0.97 (-2.64)
Infections and infestations	751	0.64 (0.59–0.68)	0.65 (150.96)	0.65 (0.60)	−0.62 (-2.29)
Respiratory, thoracic and mediastinal disorders	630	0.64 (0.59–0.69)	0.65 (127.16)	0.65 (0.60)	−0.63 (-2.29)
Surgical and medical procedures	502	1.72 (1.57–1.87)*	1.70 (145.99)	1.70 (1.55)	0.76 (-0.90)
Skin and subcutaneous Tissue disorders	427	0.34 (0.31–0.37)	0.35 (543.61)	0.35 (0.32)	−1.5 (-3.18)
Renal and urinary disorders	400	0.95 (0.86–1.05)	0.95 (1.06)	0.95 (0.86)	−0.07 (-1.74)
Musculoskeletal and connective tissue disorders	398	0.35 (0.31–0.38)	0.36 (479.86)	0.36 (0.33)	−1.48 (-3.14)
Neoplasms benign, malignant and unspecified (inclcysts and polyps)	331	0.49 (0.44–0.55)	0.50 (173.62)	0.50 (0.45)	−1.01 (-2.67)
Vascular disorders	321	0.78 (0.70–0.87)	0.78 (20.19)	0.78 (0.70)	−0.36 (-2.02)
Hepatobiliary disorders	286	1.66 (1.48–1.87)*	1.65 (74.10)	1.65 (1.47)	0.72 (-0.94)
Psychiatric disorders	222	0.19 (0.16–0.21)	0.19 (788.24)	0.19 (0.17)	−2.37 (-4.03)
Cardiac disorders	219	0.49 (0.43–0.56)	0.50 (112.10)	0.50 (0.44)	−1.00 (-2.67)
Eye disorders	71	0.17 (0.13–0.21)	0.17 (290.10)	0.17 (0.14)	−2.54 (-4.21)
Product issues	55	0.15 (0.11–0.19)	0.15 (276.01)	0.15 (0.11)	−2.76 (-4.43)
Reproductive system and breast disorders	44	0.27 (0.20–0.36)	0.27 (86.25)	0.27 (0.20)	−1.88 (-3.54)
Immune system disorders	35	0.13 (0.10–0.19)	0.14 (195.63)	0.14 (0.10)	−2.88 (-4.55)
Ear and labyrinth disorders	21	0.22 (0.15–0.34)	0.2 (56.18)	0.23 (0.15)	−2.15 (-3.82)
Social circumstances	19	0.20 (0.13–0.31)	0.20 (62.17)	0.20 (0.13)	−2.34 (-4.00)
Endocrine disorders	9	0.16 (0.08–0.31)	0.1 (39.15)	0.16 (0.08)	−2.63 (-4.29)
Congenital, familial and genetic disorders	4	0.07 (0.03–0.18)	0.07 (52.06)	0.07 (0.03)	−3.90 (-5.56)
Pregnancy, puerperium and perinatal conditions	2	0.02 (0.01–0.10)	0.02 (79.60)	0.02 (0.01)	−5.38 (-7.04)

ROR, reporting odds ratio; CI, confidence interval; PRR, proportional reporting ratio; χ2 chi-squared; IC, information component; IC 02, the lower limit of 95% CI of the IC, EBGM, empirical Bayesian geometric mean; EBGM 05, the lower limit of 95% CI of EBGM. *Indicates statistically significant signals in algorithm.

### 3.3 Preferred terms signals

**FIGURE 4:**
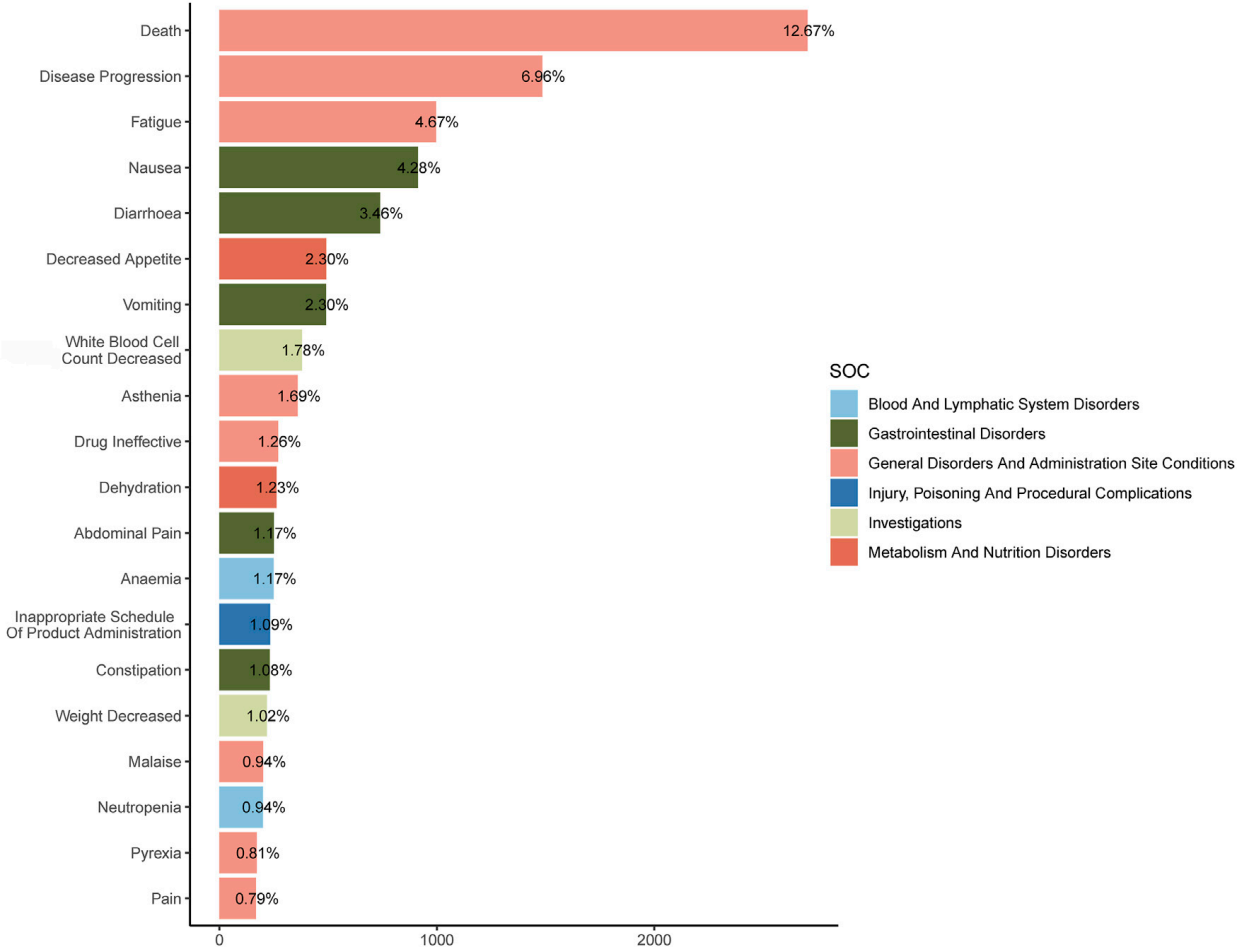
The top 20 Preferred Terms (PTs) and their affiliated System Organ Classes (SOCs).

The combined use of four algorithms successfully pinpointed 99 AEs attributed to FTD/TPI, spanning across 16 SOCs, detailed in Supplementary Table S3. Table 3 offers an overview of reported Preferred Terms (PTs) that occurred at least 10 times, featuring 50PTs across 11 SOCs. Figure 4 displays the top 20 PTs along with their respective SOCs. The three leading PTs are Death, Disease Progression, and Fatigue, all categorized under General Disorders and Administration Site Conditions. In the blood and lymphatic system disorders, gastrointestinal disorders, and general disorders and administration Site Conditions, our findings were largely consistent with previously reported PTs from previous studies. Critically, our data mining revealed several significant AEs not listed in FTD/TPI product labeling, such as iron deficiency, intestinal obstruction, ascites, small intestinal obstruction, ileus, intestinal perforation, intra-abdominal fluid collection, obstruction, hepatic failure, cholangitis, product dose omission in error, stoma site haemorrhage, carcinoembryonic antigen increased, blood iron decreased, blood magnesium decreased, dehydration, product distribution issue, product packaging quantity issue, hydronephrosis, ureteric obstruction, transfusion, stent placement and radiotherapy. More AEs were identified in our analysis, which emphasize and strengthen the overall understanding of the safety of FTD/TPI.

**TABLE 3:** Signal strength of reports of Trifluridine/Tipiracil at the Preferred Terms (PTs) level in FAERS database.

SOC	Preferred terms (PTs)	Cases reporting PT	ROR (95% two-sided CI)	PRR (χ2)	EBGM (EBGM 05)	IC(95%CI lower limit)
Blood and Lymphatic System Disorders	Anaemia	249	4.17 (3.68–4.7)	4.13 (591.74)	4.13 (3.72)	2.04 (0.38)
	Neutropenia	200	4.01 (3.49-4.61)	3.98 (446.1)	3.97 (3.53)	1.99 (0.32)
	Cytopenia	135	28.36 (23.91-33.64)	28.19 (3480.06)	27.72 (24.03)	4.79 (3.13)
	Iron Deficiency Anaemia	10	3.43 (1.85-6.38)	3.43 (17.19)	3.43 (2.04)	1.78 (0.11)
Gastrointestinal Disorders	Nausea	913	3.61 (3.38-3.86)	3.50 (1646.85)	3.49 (3.31)	1.81 (0.14)
	Diarrhoea	739	3.27 (3.04-3.52)	3.19 (1120.97)	3.19 (3.00)	1.67 (0.01)
	Vomiting	490	3.36 (3.07-3.68)	3.31 (792.39)	3.3 (3.06)	1.72 (0.06)
	Abdominal Pain	250	3.38 (2.99-3.83)	3.35 (413.7)	3.35 (3.02)	1.74 (0.08)
	Intestinal Obstruction	113	9.07 (7.53-10.92)	9.03 (802.48)	8.98 (7.69)	3.17 (1.50)
	Ascites	78	8.32 (6.66-10.39)	8.29 (497.71)	8.25 (6.85)	3.04 (1.38)
	Small Intestinal Obstruction	40	10.32 (7.56-14.09)	10.30 (333.92)	10.24 (7.9)	3.36 (1.69)
	Ileus	18	5.15 (3.24-8.18)	5.15 (59.97)	5.13 (3.49)	2.36 (0.69)
	Intestinal Perforation	14	4.02 (2.38-6.80)	4.02 (31.72)	4.02 (2.59)	2.01 (0.34)
	Intra-Abdominal Fluid Collection	11	12.35 (6.82-22.35)	12.34 (113.78)	12.25 (7.46)	3.62 (1.95)
	Gastrointestinal Toxicity	10	6.22 (3.34-11.58)	6.22 (43.65)	6.2 (3.69)	2.63 (0.97)
General Disorders and Administration Site Conditions	Death	2705	10.00 (9.60-10.41)	8.86 (19022.37)	8.81 (8.52)	3.14 (1.47)
	Disease Progression	1485	41.06 (38.93-43.31)	38.27 (52747.57)	37.41 (35.77)	5.23 (3.56)
	Fatigue	996	3.62 (3.40-3.86)	3.5 (1798.85)	3.49 (3.31)	1.81 (0.14)
	Terminal State	17	6.92 (4.30-11.15)	6.92 (85.73)	6.89 (4.63)	2.79 (1.12)
	Obstruction	15	10.20 (6.14-16.95)	10.19 (123.59)	10.13 (6.63)	3.34 (1.67)
	Performance Status Decreased	11	8.31 (4.60-15.03)	8.31 (70.36)	8.27 (5.04)	3.05 (1.38)
Hepatobiliary Disorders	Jaundice	47	6.71 (5.04-8.94)	6.70 (226.85)	6.67 (5.25)	2.74 (1.07)
	Hepatic Failure	40	4.69 (3.44-6.4)	4.69 (115.7)	4.68 (3.61)	2.23 (0.56)
	Biliary Obstruction	21	21.53 (13.99-33.12)	21.51 (405.26)	21.24 (14.81)	4.41 (2.74)
	Hyperbilirubinaemia	16	4.78 (2.92-7.81)	4.77 (47.61)	4.76 (3.16)	2.25 (0.59)
	Cholangitis	12	6.07 (3.44-10.7)	6.06 (50.56)	6.05 (3.76)	2.60 (0.93)
Injury, Poisoning and Procedural Complications	Product Dose Omission In Error	46	5.82 (4.36-7.78)	5.81 (182.76)	5.8 (4.55)	2.54 (0.87)
	Stoma Site Haemorrhage	14	19.66 (11.6-33.31)	19.65 (244.8)	19.42 (12.49)	4.28 (2.61)
Investigations	White Blood Cell Count Decreased	380	9.93 (8.97-10.99)	9.77 (2978.72)	9.72 (8.92)	3.28 (1.61)
	Platelet Count Decreased	145	3.95 (3.36-4.65)	3.93 (316.78)	3.92 (3.42)	1.97 (0.31)
	Haemoglobin Decreased	133	4.12 (3.48-4.89)	4.10 (312.00)	4.10 (3.55)	2.03 (0.37)
	Red Blood Cell Count Decreased	94	9.51 (7.76-11.66)	9.47 (708.70)	9.43 (7.95)	3.24 (1.57)
	Neutrophil Count Decreased	84	5.93 (4.78-7.35)	5.91 (341.50)	5.89 (4.92)	2.56 (0.89)
	Full Blood Count Abnormal	65	5.44 (4.26-6.94)	5.43 (234.04)	5.41 (4.41)	2.44 (0.77)
	Blood Bilirubin Increased	50	6.8 (5.15-8.98)	6.79 (245.88)	6.76 (5.36)	2.76 (1.09)
	Carcinoembryonic Antigen Increased	35	51.39 (36.7-71.97)	51.31 (1673.25)	49.76 (37.54)	5.64 (3.97)
	Blood Iron Decreased	19	4.33 (2.76-6.79)	4.32 (48.40)	4.31 (2.96)	2.11 (0.44)
	Blood Magnesium Decreased	11	3.63 (2.01-6.57)	3.63 (20.94)	3.63 (2.21)	1.86 (0.19)
Metabolism and Nutrition Disorders	Decreased Appetite	491	6.12 (5.60-6.70)	6.01 (2048.66)	5.99 (5.55)	2.58 (0.92)
	Dehydration	262	6.64 (5.87-7.5)	6.57 (1233.48)	6.54 (5.91)	2.71 (1.04)
	Feeding Disorder	30	3.81 (2.66-5.46)	3.81 (62.04)	3.8 (2.82)	1.93 (0.26)
	Failure To Thrive	10	7.73 (4.15-14.4)	7.73 (58.32)	7.7 (4.58)	2.94 (1.28)
Nervous System Disorders	Dysgeusia	82	3.48 (2.80-4.32)	3.47 (143.77)	3.46 (2.89)	1.79 (0.13)
Product Issues	Product Distribution Issue	19	7.55 (4.81-11.84)	7.54 (107.28)	7.51 (5.15)	2.91 (1.24)
	Product Packaging Quantity Issue	15	3.89 (2.34-6.46)	3.89 (32.11)	3.88 (2.54)	1.96 (0.29)
Renal and Urinary Disorders	Hydronephrosis	20	8.76 (5.64-13.59)	8.75 (136.58)	8.71 (6.03)	3.12 (1.46)
	Ureteric Obstruction	11	28.69 (15.8-52.09)	28.68 (288.69)	28.19 (17.12)	4.82 (3.15)
Surgical and Medical Procedures	Transfusion	88	21.96 (17.79-27.11)	21.87 (1729.63)	21.59 (18.10)	4.43 (2.77)
	Stent Placement	16	7.04 (4.31-11.5)	7.03 (82.46)	7.01 (4.65)	2.81 (1.14)
	Radiotherapy	12	21.73 (12.29-38.42)	21.72 (234.07)	21.45 (13.31)	4.42 (2.75)

ROR, reporting odds ratio; CI, confidence interval; PRR, proportional reporting ratio; χ2 chi-squared; IC, information component; IC 025, the lower limit of 95% CI of the IC; EBGM, empirical Bayesian geometric mean; EBGM 05: the lower limit of 95% CI of EBGM. *Emerging findings of Trifluridine/Tipiracil associated AEs, from FAERS, database.

### 3.4 Subgroup analysis of signals for preferred terms

In the analysis of the two age subgroups, 18-65 years and over 65 years (<18 years subgroup excluded due to under-reporting of cases), Vomiting, Abdominal Pain, Platelet Count Decreased, and Intestinal Obstruction were notably prevalent in the younger cohort, while Malaise and Haemoglobin Decreased were more common in the older group (Supplementary Tables S4, S5). Together, such information would be critical for more refined clinical management, guiding clinical decision makers to tailor treatments to the characteristics of specific subgroups.

We analyzed the top 15 Preferred Terms (PTs) for both male and female subgroups and found that the first nine PTs were identical in both groups. However, Anaemia, Constipation, Neutropenia, Malaise, and Platelet Count Decreased were more common in males, while Dehydration, Weight Decreased, and Alopecia were ranked higher in females (Supplementary Tables S6, S7).

### 3.5 Time to onset of adverse events

**FIGURE 5:**
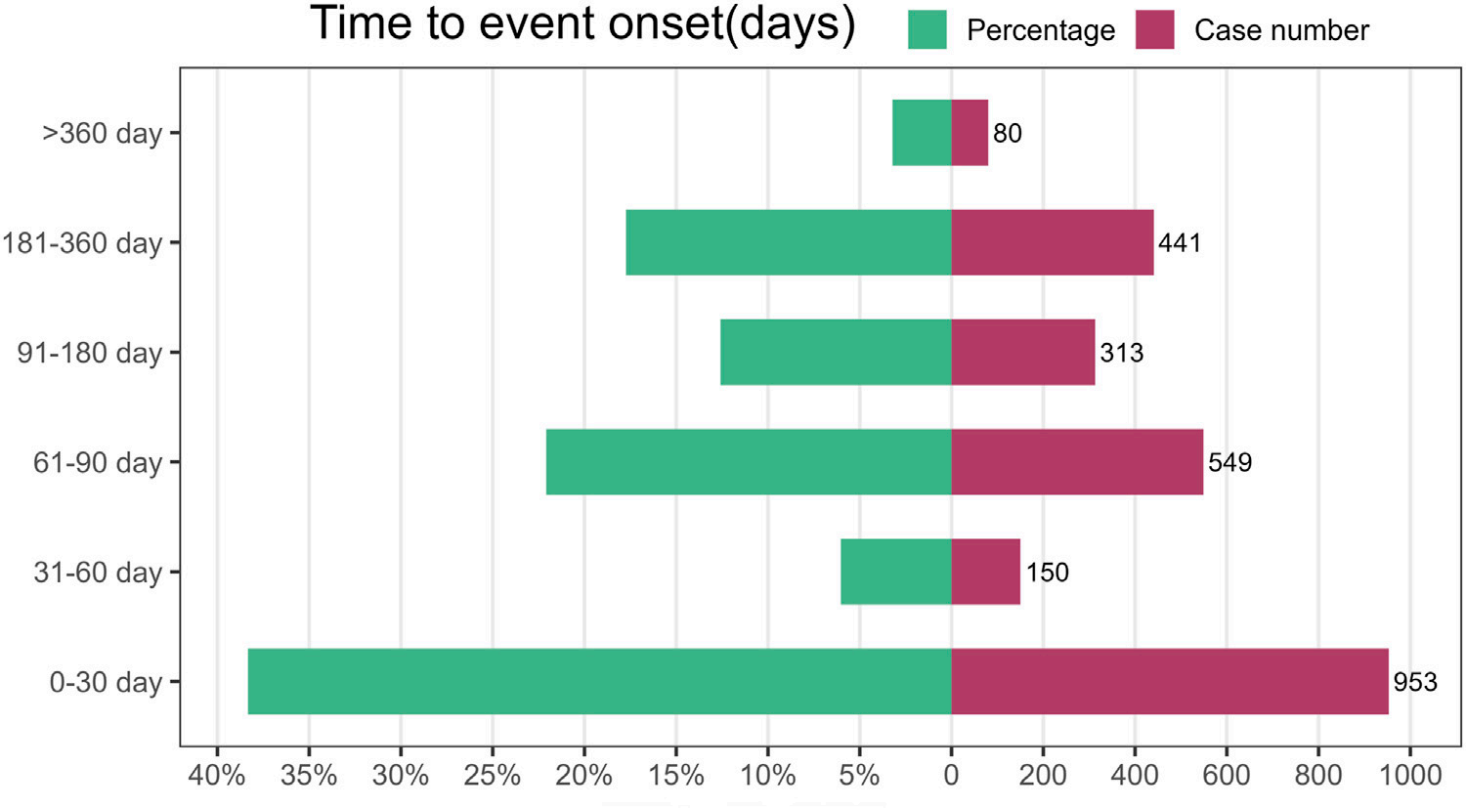
Time to onset of adverse events (AEs) associated with FTD/TPI.

The database provided detailed information on the timing of AEs linked to FTD/TPI use. Among all reports, 2,487 offered detailed and accurate timelines for when these incidents occurred. The AEs had a median onset time of 44 days, with an IQR of 20–97 days Figure 5 illustrates that a significant portion of adverse events (AEs), specifically 953 (38.33%), occurred within the first month of starting FTD/TPI, according to the AE onset time distribution. The time to treatment greater than 1 year was the lowest reported rate. The lowest rates were reported for treatment durations greater than 1 year (N = 80, 3.22%). There was no pattern in the reported rates for the remaining dosing times.

## 4 Discussion

Earlier research on FTD/TPI largely targeted its mechanism, clinical trials, and literature reviews, with minimal focus on contemporary real-world studies. To the best of our knowledge, this research utilizing the FAERS database to explore FTD/TPI related AEs represents the broadest pharmacovigilance study to date. It delivers an in-depth and systematic examination of global reports on adverse events associated with FTD/TPI as recorded in FAERS. The objective is to unearth novel and significant adverse reactions, aid in updating the product’s summary of characteristics, and support evidence-based clinical usage.

Following the baseline profile information, men experienced adverse reactions to FTD/TPI more frequently (57.10%) than women (42.00%), possibly due to the higher incidence of colorectal cancer in males, which may lead to increased medication usage. This aligns with the epidemiology of colorectal cancer (Kocarnik et al., 2022). In addition, we found that more AEs were noted in the over 65 age group (37.67%) compared to 18-65 age group (36.90%). This trend underscores the age-specific vulnerability and the critical period for heightened surveillance and management of AEs in the elderly demographic of colorectal cancer patients. One issue we have to note is that death was categorized as the most severe AEs in the report on outcomes, with a percentage of 42.02%. Although this may be due to the condition’s severity or its progression, given FTD/TPI’s use for fluoropyrimidine-resistant metastatic colorectal cancer, the significance of such a high rate of AEs cannot be overlooked. Attention is particularly warranted for those in late-stage or with compromised health, concerning the adjustment of TFTD/TPI dosage. It is notable that a substantial proportion (42.50%) of adverse reaction reports for TFTD/TPI were submitted by consumers, not healthcare professionals. This trend could indicate a higher propensity among patients to report side effects directly or suggest underreporting by healthcare workers. Given that a vast majority of these reports come from the U.S. (82.00%), it may also highlight specific regional or cultural reporting habits. Further analysis is necessary to ascertain if regional or cultural factors significantly influence this reporting pattern.

Disproportionality analysis identified significant AEs associated with TFTD/TPI across various SOCs, such as general disorders and administration site conditions, gastrointestinal disorders, investigations, metabolism and nutrition disorders, blood and lymphatic system disorders, surgical and medical procedures, hepatobiliary disorders (Figure 2). This highlights the need for careful monitoring in these areas during TFTD/TPI treatment. Aside from surgical and medical procedures, several SOCs are frequently reported in clinical trials (Zaniboni et al., 2021; Taieb et al., 2023). This study identified positive signals for specific adverse reactions, such as myelosuppression, gastrointestinal toxicity, systemic weakness/fatigue, decreased appetite, and increased blood bilirubin, all mentioned in drug labels, thus reinforcing the reliability of our findings. However, it also revealed that certain AEs within some SOCs, such as obstruction, decreased blood iron, decreased blood magnesium, dehydration, feeding disorder, failure to thrive, need for transfusion or stent placement, radiotherapy, biliary obstruction, hyperbilirubinemia, and cholangitis, are not listed on the drug’s label.

Within the SOC of General disorders and administration site conditions, notable associations were observed with death (n = 2705), disease progression (n = 1485), and fatigue (n = 996), highlighting these as significant adverse events in the treatment context. Currently, there are no studies explicitly indicating that the use of TFTD/TPI increases patient mortality. This includes two major clinical trials where, despite 5 and 1 reported deaths in the TFTD/TPI groups respectively, the adverse events leading to death were not considered related to the study drug (Mayer et al., 2015; Xu et al., 2018). WJOG14520G is a study on the combination of FTD/TPI and Bevacizumab for treating mCRC in frail patients, with a median age of 79 years in the cohort, 65% of patients aged ≥75 years, 26% having severe comorbidities, and 20% with poor performance status. The study observed no treatment-related deaths (Kito et al., 2024). Other research has indicated that disease progression is the most common cause reported in fatal adverse events among patients (Kröning et al., 2023). It might be unreasonable to judge solely by signals whether TFTD/TPI causes tumor metastasis and progression, as previously discussed. Nonetheless, clinicians should still discern if patient death or disease progression is related to TFTD/TPI use. Despite this, other AEs induced by TFTD/TPI treatment deserve attention since they significantly exceed those caused by placebo and/or best supportive care (Huang et al., 2024). The incidence of overall AEs is higher with combination therapy compared to using TFTD/TPI alone (Kagawa et al., 2023). Numerous studies have shown that neutropenia, anemia, thrombocytopenia, fatigue, nausea, decreased appetite, diarrhea, vomiting, and abdominal pain are among the most common adverse reactions or laboratory abnormalities associated with TFTD/TPI(Prager et al., 2023; Taieb et al., 2023), consistent with our observations. Neutropenia is the most common hematological adverse reaction, yet studies suggest that neutropenia serves as a useful prognostic indicator for the efficacy of TFTD/TPI treatment (Nose et al., 2020; Yoshino et al., 2020; Domínguez Senín et al., 2023). Additionally, we identified other signals, such as intestinal obstruction and perforation, not specifically linked to TFTD/TPI use alone. However, combination treatments like TFTD/TPI with Bevacizumab, commonly used for metastatic colorectal cancer, may contribute (Kagawa et al., 2023; Prager et al., 2023; Kuboki et al., 2024). Reports exist of Bevacizumab causing gastrointestinal perforation (Qi et al., 2014; Wichelmann et al., 2021) and obstruction (Zaniboni et al., 2021). When treating both gastrointestinal and non-gastrointestinal cancers. Patients with a history of chemotherapy for gynecological cancer who use Bevacizumab have an increased risk of gastrointestinal perforation, especially those with more than three chemotherapy cycles and a history of intestinal resection, facing even higher risks of perforation (Matsumiya et al., 2020). Another meta-analysis on the increased risk of gastrointestinal perforation in cancer patients treated with Bevacizumab showed a significantly higher risk in colorectal cancer patients (Qi et al., 2014). Clinicians should be aware of the potential increased risk of gastrointestinal perforation in patients treated with TFTD/TPI and Bevacizumab for colorectal cancer and recommend close monitoring. Additionally, we reported other adverse events related to obstruction including ureteral obstruction and hydronephrosis.

We conducted subgroup analyses based on age and gender for reported adverse reactions. The top 20 adverse reactions reported were 3,892 in the <65 years group and 4,588 in the ≥65 years group, with the same first two adverse reactions in both groups, death and disease progression. However, vomiting, decreased platelet count, and intestinal obstruction were more reported in the <65 group, while malaise and decreased haemoglobin were more prevalent in the ≥65 group.

Subgroup analysis by gender revealed that males reported a higher total of the top 20 adverse reactions (5999 items) compared to females (4839 items). However, the names of the specific AEs within the top 10 rankings were the same for both groups.

The EROTAS-R study on TAS-102’s effects and risk factors in metastatic CRC patients found that gender and age are not predictors of adverse event (AE) occurrence (Yoshida et al., 2023). Another study analyzing risk factors for nausea and vomiting in metastatic colorectal cancer patients treated with FTD/TPI and Bevacizumab also concluded that age and gender are not risk factors for these conditions (Prejac et al., 2024). An American study on the safety of FTD/TPI in elderly and younger patients with metastatic colorectal cancer showed consistent results between the two age groups. Both the elderly (≥65 years old) and younger (<65 years old) subgroups experienced similar proportions of Grade≥3 AEs (Mayer et al., 2018). The TERRA study indicated that for patients under 65, the risk of death in the FTD/TPI group was 0.90 (95% CI 0.69-1.18) compared to the placebo group, while for older patients, the risk was significantly lower at 0.45 (95% CI 0.28-0.74) (Xu et al., 2018). A multicenter retrospective study involving an elderly patient subgroup analysis showed no difference in common hematological and gastrointestinal adverse reactions between the <70 and ≥70 older age groups. However, neutropenia, febrile neutropenia, and asthenia were significantly higher in the group aged ≥75 compared to those <75 years old (Conti et al., 2023). Further findings show no disparity in overall age-related adverse event (AE) rates between FTD/TPI and placebo groups. Patients over 65 tend to report more grade ≥3 and severe AEs than their younger counterparts. The group aged 75 and above showed a marginally higher occurrence of treatment-related AEs compared to those aged 65-74 (91.7% vs 83.6%), yet the incidence of Grade ≥3 AEs was nearly identical (75% vs 74.8%), with severe AEs reported at 33.3% and 30.8%, respectively (Victorino et al., 2023). The PRECONNECT study, a multicenter clinical trial on the safety and efficacy of FTD/TPI, found that the incidence of grade ≥3 treatment-related adverse reactions was 79.6% in patients over 70 years old and 72.5% in those aged 70 or younger (Bachet et al., 2020). There are variations in the results across reports. Overall, our study results offer insights into side effects related to gender and age. Although these findings require further validation, they provide improved guidance for drug monitoring. We should also emphasize the significance of sociodemographic factors such as age and gender to enhance the safe use of FTD/TPI.

The time-to-onset analysis indicated that adverse events associated with TFTD/TPI typically emerged 44 days post-treatment initiation, with a significant number of cases (n = 953, 38.33%) occurring within the first month of TFTD/TPI use. This underscores the critical period shortly after treatment commencement for monitoring adverse reactions.

## 5 Limitations

While the study benefits from large-scale real-world data and advanced analytics, limitations exist due to reliance on voluntary reporting, potentially leading to bias and incomplete data. For example, reports from consumers might lack the reliability and detail of those from healthcare professionals, and there may be a reporting bias towards regions with more frequent reporting (Jiang et al., 2024). Given TFTD/TPI’s extended market presence, including its availability as a generic drug in some countries, which may alter absorption rates and its safety profile, the accurate incidence of AEs cannot be ascertained solely from FAERS data. Disproportionality analysis, while highlighting signal strength statistically, does not measure risk or establish causality, offering only a signal estimation (Zou et al., 2024). Future clinical research is essential to establish a definitive cause-and-effect link. Despite its limitations, our findings serve as a crucial resource for healthcare practitioners to enhance patient follow-up and vigilance regarding TFTD/TPI-related adverse reactions.

## 6 Conclusion

Our pharmacovigilance analysis of the FAERS database provides a comprehensive and systematic overview of TFTD/TPI’s safety signals and timing of AEs. We’ve identified both new and unexpected significant AEs like iron deficiency, intestinal obstruction, and ureteric obstruction, alongside common AEs such as myelosuppression and gastrointestinal toxicity. Continuous monitoring and risk identification for these AEs across all populations are recommended. Yet, cohort studies and long-term clinical research are necessary to validate these findings and further elucidate TFTD/TPI’s safety profile.

## Data Availability

The original contributions presented in the study are included in the article/Supplementary Material, further inquiries can be directed to the corresponding authors.

## Supplementary material

SUPPLEMENTARY FIGURE S1 Clinical characteristics of FTD/TPI-associated AEs.

SUPPLEMENTARY TABLE S1 Four-compartment table of drugs and adverse reactions.

SUPPLEMENTARY TABLE S2 Four algorithmic formulas and criteria.

SUPPLEMENTARY TABLE S3 Signal strength of reports of Trifluridine/Tipiracil at the Preferred Terms (PTs) level in FAERS database.

SUPPLEMENTARY TABLE S4 Signal strength of reports of Trifluridine/Tipiracil at the Preferred Terms (PTs) level in FAERS database (<65 years old).

SUPPLEMENTARY TABLE S5 Signal strength of reports of Trifluridine/Tipiracil at the Preferred Terms (PTs) level in FAERS database (≥65years old).

SUPPLEMENTARY TABLE S6 Signal strength of reports of Trifluridine/Tipiracil at the Preferred Terms (PTs) level in FAERS database (male).

SUPPLEMENTARY TABLE S7 Signal strength of reports of Trifluridine/Tipiracil at the Preferred Terms (PTs) level in FAERS database (female).
