# Supplementary material for: Signal mining and analysis of trifluridine/tipiracil adverse events based on real-world data from the FAERS database
Source: Front Pharmacol. 2024 Jul 23;15:1399998. doi: 10.3389/fphar.2024.1399998 (PMC11301057; doi:10.3389/fphar.2024.1399998)
Supplement: Supplementary file 1 [file Table1.docx]

| **Supplementary Table 1**. Four-compartment table of drugs and adverse reactions | | | |
| --- | --- | --- | --- |
| drug | FTD/TPI associated AEs | Non-FTD/TPI associated AEs | total |
| FTD/TPI | a | b | a+b |
| Non-FTD/TPI | c | d | c+d |
| total | a+c | b+d | a+b+c+d |

AEs, adverse events. a represents the count of instances where a specific adverse event was observed following the administration of FTD/TPI. B denotes the instances where FTD/TPI was administered but the specific adverse event did not occur. c accounts for the occurrences of the specific adverse event in the absence of FTD/TPI usage. d indicates the number of cases where neither FTD/TPI was used nor the adverse event took place.
