# Supplementary material for: Signal mining and analysis of trifluridine/tipiracil adverse events based on real-world data from the FAERS database
Source: Front Pharmacol. 2024 Jul 23;15:1399998. doi: 10.3389/fphar.2024.1399998 (PMC11301057; doi:10.3389/fphar.2024.1399998)
Supplement: Supplementary file 2 [file Table7.docx]

| **Supplementary Table 7.** Signal strength of reports of Trifluridine/Tipiracil at the Preferred Terms (PTs) level in FAERS database（female） | | | | |
| --- | --- | --- | --- | --- |
| **PT** | **N** | **ROR** | **(95%Cl) Lower** | **(95%Cl) Upper** |
| Death | 1038 | 12.15 | 11.39 | 12.96 |
| Disease Progression | 616 | 48.24 | 44.42 | 52.39 |
| Nausea | 476 | 3.56 | 3.25 | 3.91 |
| Fatigue | 451 | 3.41 | 3.1 | 3.75 |
| Diarrhoea | 346 | 3.26 | 2.93 | 3.63 |
| Vomiting | 277 | 3.77 | 3.34 | 4.25 |
| Decreased Appetite | 216 | 6.27 | 5.48 | 7.18 |
| White Blood Cell Count Decreased | 177 | 9.04 | 7.79 | 10.49 |
| Asthenia | 162 | 2.92 | 2.5 | 3.41 |
| Dehydration | 130 | 7.47 | 6.28 | 8.88 |
| Abdominal Pain | 114 | 3.21 | 2.67 | 3.86 |
| Weight Decreased | 111 | 2.75 | 2.28 | 3.31 |
| Inappropriate Schedule Of Product Administration | 111 | 2.49 | 2.06 | 3 |
| Constipation | 101 | 3.04 | 2.5 | 3.7 |
| Anaemia | 100 | 4.12 | 3.39 | 5.02 |
| Alopecia | 91 | 1.83 | 1.49 | 2.24 |
| Neutropenia | 88 | 4.91 | 3.98 | 6.06 |
| Pyrexia | 83 | 1.74 | 1.4 | 2.16 |
| Abdominal Pain Upper | 80 | 2.28 | 1.83 | 2.84 |
| Haemoglobin Decreased | 71 | 5.48 | 4.33 | 6.92 |

ROR: reporting odds ratio, CI: confidence interval.
