# Supplementary material for: Signal mining and analysis of trifluridine/tipiracil adverse events based on real-world data from the FAERS database
Source: Front Pharmacol. 2024 Jul 23;15:1399998. doi: 10.3389/fphar.2024.1399998 (PMC11301057; doi:10.3389/fphar.2024.1399998)
Supplement: Supplementary file 3 [file Table2.docx]

**Supplementary Table 2. Four algorithmic formulas and criteria**

| Algorithms | Equation | Criteria |
| --- | --- | --- |
| ROR | ROR=ad/bc, 95%CI =eln(ROR)±1.96(1/a+1/b+1/c+1/d)^0.5 | lower limit of 95% CI>1, a≥3 |
| PRR | PRR=[a(c+d)]/[c (a+b)] χ²=[(ad-bc)^2](a+b+c+d)/[(a+b)(c+d)(a+c)(b+d)] | PRR≥2, χ²≥4, a≥3 |
| BCPNN | IC= log2a (a+b +c+ d)/[(a+c)(a+b)],  95%CI =E (IC) ±2V(IC)^0.5 | IC025>0, a>0 |
| MGPS | EBGM=a(a+b+c+d)/(a+c)(a+b), EBGM05=eln(EBGM)-1.64(1/a+1/b+1/c+1/d)^0.5 | EBGM05>2, a>0 |

Equation: a, number of reports containing both the target drug and the target adverse drug reaction; b, number of reports containing other adverse drug reactions of the target drug; c, number of reports containing the target adverse drug reaction of other drugs; d, number of reports containing other drugs and other adverse drug reactions. The MGPS employs an empirical Bayesian approach, whereby a prior distribution is obtained by maximum likelihood estimates, and the prior and likelihood are subsequently combined to obtain a posterior distribution. The fifth percentile of the posterior distribution is denoted by “EBGM05” and is interpreted as the one-sided 95% confidence lower bound for the EBGM. Abbreviations: 95% CI, 95% confidence interval; N, the number of reports; χ2, chi-squared; IC, information component; IC025, the lower limit of the 95% CI of the IC; E (IC), the IC expectations; V (IC), the variance of IC; EBGM, empirical Bayesian geometric mean; EBGM05, empirical Bayesian geometric mean lower 95% CI for the posterior distribution.
