# Supplementary material for: Signal mining and analysis of trifluridine/tipiracil adverse events based on real-world data from the FAERS database
Source: Front Pharmacol. 2024 Jul 23;15:1399998. doi: 10.3389/fphar.2024.1399998 (PMC11301057; doi:10.3389/fphar.2024.1399998)
Supplement: Supplementary file 4 [file Table5.docx]

| **Supplementary Table 5.** Signal strength of reports of Trifluridine/Tipiracil at the Preferred Terms (PTs) level in FAERS database（≥65years old） | | | | |
| --- | --- | --- | --- | --- |
| **PT** | **N** | **ROR** | **(95%Cl) lower** | **Upper** |
| Death | 1020 | 5.67 | 5.31 | 6.06 |
| Disease Progression | 480 | 26.27 | 23.93 | 28.84 |
| Fatigue | 438 | 3.54 | 3.22 | 3.9 |
| Diarrhoea | 382 | 3.04 | 2.74 | 3.37 |
| Nausea | 329 | 3.10 | 2.78 | 3.47 |
| Decreased Appetite | 226 | 4.29 | 3.76 | 4.9 |
| White Blood Cell Count Decreased | 212 | 9.27 | 8.08 | 10.63 |
| Asthenia | 196 | 2.47 | 2.14 | 2.84 |
| Vomiting | 183 | 2.93 | 2.53 | 3.4 |
| Anaemia | 148 | 3.25 | 2.76 | 3.82 |
| Dehydration | 135 | 4.86 | 4.10 | 5.77 |
| Inappropriate Schedule Of Product Administration | 114 | 3.21 | 2.67 | 3.86 |
| Neutropenia | 107 | 4.04 | 3.34 | 4.89 |
| Constipation | 102 | 2.3 | 1.97 | 2.91 |
| Weight Decreased | 98 | 1.94 | 1.59 | 2.37 |
| Malaise | 92 | 1.23 | 1.00 | 1.51 |
| Abdominal Pain | 88 | 2.83 | 2.30 | 3.50 |
| Abdominal Pain Upper | 80 | 2.67 | 2.14 | 3.33 |
| Pyrexia | 79 | 1.53 | 1.23 | 1.91 |
| Haemoglobin Decreased | 79 | 3.64 | 2.91 | 4.54 |

ROR: reporting odds ratio, CI: confidence interval.
