# Supplementary material for: Signal mining and analysis of trifluridine/tipiracil adverse events based on real-world data from the FAERS database
Source: Front Pharmacol. 2024 Jul 23;15:1399998. doi: 10.3389/fphar.2024.1399998 (PMC11301057; doi:10.3389/fphar.2024.1399998)
Supplement: Supplementary file 5 [file Table3.docx]

| **Supplementary Table 3. Signal strength of reports of Trifluridine/Tipiracil at the Preferred Terms (PTs) level in FAERS database** | | | | | | |
| --- | --- | --- | --- | --- | --- | --- |
| **SOC** | **Preferred terms (PTs)** | **Cases Reporting**  **PT** | **ROR(95% two-sided CI)** | **PRR(χ2)** | **EBGM**  **(EBGM 05)** | **IC(95%CI lower limit)** |
| Blood and Lymphatic System Disorders | Anaemia | 249 | 4.17 (3.68 - 4.7) | 4.13(591.74) | 4.13(3.72) | 2.04(0.38) |
|  | Neutropenia | 200 | 4.01(3.49-4.61) | 3.98(446.1) | 3.97(3.53) | 1.99(0.32) |
|  | Cytopenia | 135 | 28.36(23.91-33.64) | 28.19(3480.06) | 27.72(24.03) | 4.79(3.13) |
|  | Iron Deficiency Anaemia | 10 | 3.43(1.85-6.38) | 3.43(17.19) | 3.43(2.04) | 1.78(0.11) |
| Cardiac Disorders | Cardiopulmonary Failure | 7 | 7.09(3.37-14.89) | 7.08(36.42) | 7.06(3.79) | 2.82(1.15) |
| Gastrointestinal Disorders | Nausea | 913 | 3.61(3.38-3.86) | 3.50(1646.85) | 3.49(3.31) | 1.81(0.14) |
|  | Diarrhoea | 739 | 3.27(3.04-3.52) | 3.19(1120.97) | 3.19(3.00) | 1.67(0.01) |
|  | Vomiting | 490 | 3.36(3.07-3.68) | 3.31(792.39) | 3.3(3.06) | 1.72(0.06) |
|  | Abdominal Pain | 250 | 3.38(2.99-3.83) | 3.35(413.7) | 3.35(3.02) | 1.74(0.08) |
|  | Intestinal Obstruction | 113 | 9.07(7.53-10.92) | 9.03(802.48 ) | 8.98(7.69) | 3.17(1.50) |
|  | Ascites | 78 | 8.32(6.66-10.39) | 8.29(497.71) | 8.25(6.85) | 3.04(1.38) |
|  | Small Intestinal Obstruction | 40 | 10.32(7.56-14.09) | 10.30(333.92) | 10.24(7.9) | 3.36(1.69) |
|  | Ileus | 18 | 5.15(3.24-8.18) | 5.15(59.97) | 5.13(3.49) | 2.36(0.69) |
|  | Intestinal Perforation | 14 | 4.02(2.38-6.80) | 4.02(31.72) | 4.02(2.59) | 2.01(0.34) |
|  | Intra-Abdominal Fluid Collection | 11 | 12.35(6.82-22.35) | 12.34(113.78) | 12.25(7.46) | 3.62(1.95) |
|  | Gastrointestinal Toxicity | 10 | 6.22(3.34-11.58) | 6.22(43.65) | 6.2(3.69) | 2.63(0.97) |
|  | Large Intestinal Obstruction | 7 | 8.49(4.04-17.86) | 8.49(46.03) | 8.45(4.54) | 3.08(1.41) |
|  | Proctalgia | 7 | 3.45(1.64-7.24) | 3.45(12.15) | 3.44(1.85) | 1.78(0.12) |
|  | Oesophageal Varices Haemorrhage | 6 | 8.52(3.82-19) | 8.52(39.6) | 8.48(4.33) | 3.08(1.42) |
|  | Proctitis | 6 | 6.23(2.79-13.88) | 6.22(26.21) | 6.2(3.17) | 2.63(0.97) |
|  | Malignant Ascites | 5 | 23.22(9.6-56.14) | 23.21(104.78) | 22.9(10.94) | 4.52( 2.84) |
|  | Large Intestinal Haemorrhage | 5 | 6.51(2.71-15.68) | 6.51(23.24) | 6.49(3.11) | 2.7(1.03) |
|  | Neutropenic Colitis | 4 | 5.39(2.02-14.38) | 5.39(14.25) | 5.37(2.36) | 2.43(0.76) |
|  | Rectal Perforation | 3 | 18.31(5.87-57.14) | 18.31(48.54) | 18.11(6.99) | 4.18(2.5) |
|  | Subileus | 3 | 4.90(1.58-15.21) | 4.90(9.28) | 4.89(1.89) | 2.29(0.62) |
|  | Small Intestinal Perforation | 3 | 5.18(1.67-16.08) | 5.17(10.07) | 5.16(2.00) | 2.37(0.70) |
|  | Oesophageal Haemorrhage | 3 | 6.99(2.25-21.71) | 6.98(15.32) | 6.96(2.69) | 2.80(1.13) |
|  | Enterocutaneous Fistula | 3 | 10.58(3.4-32.92) | 10.58(25.84) | 10.51(4.07) | 3.39(1.72) |
|  | Duodenal Ulcer Haemorrhage | 3 | 3.28(1.06-10.17) | 3.28(4.74) | 3.27(1.27) | 1.71(0.04) |
|  | Gastrointestinal Obstruction | 3 | 3.26(1.05-10.11) | 3.25(4.68) | 3.25(1.26) | 1.70(0.03) |
| General Disorders and Administration SiteConditions | Death | 2705 | 10.00(9.60-10.41) | 8.86(19022.37) | 8.81(8.52) | 3.14(1.47) |
|  | Disease Progression | 1485 | 41.06(38.93-43.31) | 38.27(52747.57) | 37.41(35.77) | 5.23(3.56) |
|  | Fatigue | 996 | 3.62(3.40-3.86) | 3.5(1798.85) | 3.49(3.31) | 1.81(0.14) |
|  | Terminal State | 17 | 6.92(4.30-11.15) | 6.92(85.73) | 6.89(4.63) | 2.79(1.12) |
|  | Obstruction | 15 | 10.20(6.14-16.95) | 10.19(123.59) | 10.13(6.63) | 3.34(1.67) |
|  | Performance Status Decreased | 11 | 8.31(4.60-15.03) | 8.31(70.36) | 8.27(5.04) | 3.05(1.38) |
|  | Pelvic Mass | 4 | 17.28(6.45-46.29) | 17.28(60.69) | 17.1(7.50) | 4.10(2.42) |
|  | Organ Failure | 3 | 3.34(1.08-10.36) | 3.34(4.9) | 3.33(1.29) | 1.74(0.07) |
| Hepatobiliary Disorders | Jaundice | 47 | 6.71(5.04-8.94) | 6.70(226.85) | 6.67(5.25) | 2.74(1.07) |
|  | Hepatic Failure | 40 | 4.69(3.44-6.4) | 4.69(115.7) | 4.68(3.61) | 2.23(0.56) |
|  | Biliary Obstruction | 21 | 21.53(13.99-33.12) | 21.51(405.26) | 21.24(14.81) | 4.41(2.74) |
|  | Hyperbilirubinaemia | 16 | 4.78(2.92-7.81) | 4.77(47.61) | 4.76(3.16) | 2.25(0.59) |
|  | Cholangitis | 12 | 6.07(3.44-10.7) | 6.06(50.56) | 6.05(3.76) | 2.60(0.93) |
|  | Hepatic Pain | 9 | 6.18(3.21-11.89) | 6.18(38.91) | 6.16(3.56) | 2.62(0.96) |
|  | Cholecystitis Acute | 6 | 4.07(1.83-9.08) | 4.07(13.87) | 4.06(2.08) | 2.02(0.36) |
|  | Jaundice Cholestatic | 5 | 5.13(2.13-12.33) | 5.12(16.55) | 5.11(2.45) | 2.35(0.69) |
|  | Portal Hypertension | 3 | 3.30(1.06-10.26) | 3.30(4.81) | 3.3(1.28) | 1.72(0.05) |
|  | Gallbladder Enlargement | 3 | 8.79(2.83-27.34) | 8.79(20.59) | 8.75(3.38) | 3.13(1.46) |
| Infections and Infestations | Abdominal Infection | 5 | 5.48(2.28-13.18) | 5.48(18.23) | 5.46(2.62) | 2.45(0.78) |
|  | Escherichia Urinary Tract Infection | 5 | 3.57(1.48-8.59) | 3.57(9.23) | 3.56(1.71) | 1.83(0.17) |
|  | Abdominal Abscess | 5 | 3.56(1.48-8.57) | 3.56( 9.19 ) | 3.56(1.71) | 1.83(0.16) |
|  | Escherichia Sepsis | 3 | 3.31(1.06-10.26) | 3.31(4.82) | 3.3(1.28) | 1.72(0.06) |
|  | Campylobacter Gastroenteritis | 3 | 13.54(4.35-42.19) | 13.54(34.55) | 13.43(5.19) | 3.75(2.07) |
|  | Biliary Tract Infection | 3 | 13.06(4.19-40.7) | 13.06(33.15) | 12.97(5.01) | 3.70(2.02) |
| Injury, Poisoning and Procedural Complications | Product Dose Omission In Error | 46 | 5.82(4.36-7.78) | 5.81(182.76) | 5.8(4.55) | 2.54(0.87) |
|  | Stoma Site Haemorrhage | 14 | 19.66(11.6-33.31) | 19.65(244.8) | 19.42(12.49) | 4.28(2.61) |
|  | Gastrointestinal Stoma Complication | 4 | 6.91(2.59-18.45) | 6.91(20.12) | 6.88(3.03) | 2.78(1.11) |
|  | Procedural Vomiting | 3 | 13.28(4.26-41.37) | 13.28(33.78) | 13.18(5.09) | 3.72(2.05) |
| Investigations | White Blood Cell Count Decreased | 380 | 9.93(8.97-10.99) | 9.77(2978.72) | 9.72(8.92) | 3.28( 1.61) |
|  | Platelet Count Decreased | 145 | 3.95(3.36-4.65) | 3.93(316.78) | 3.92(3.42) | 1.97(0.31) |
|  | Haemoglobin Decreased | 133 | 4.12(3.48-4.89) | 4.10(312.00) | 4.10(3.55) | 2.03(0.37) |
|  | Red Blood Cell Count Decreased | 94 | 9.51(7.76-11.66) | 9.47(708.70 ) | 9.43(7.95) | 3.24(1.57) |
|  | Neutrophil Count Decreased | 84 | 5.93(4.78-7.35) | 5.91(341.50) | 5.89(4.92) | 2.56(0.89) |
|  | Full Blood Count Abnormal | 65 | 5.44(4.26-6.94) | 5.43(234.04) | 5.41(4.41) | 2.44(0.77) |
|  | Blood Bilirubin Increased | 50 | 6.8(5.15-8.98) | 6.79(245.88) | 6.76(5.36) | 2.76(1.09) |
|  | Carcinoembryonic Antigen Increased | 35 | 51.39(36.7-71.97) | 51.31(1673.25) | 49.76(37.54) | 5.64(3.97) |
|  | Blood Iron Decreased | 19 | 4.33(2.76-6.79) | 4.32(48.40) | 4.31(2.96) | 2.11(0.44) |
|  | Blood Magnesium Decreased | 11 | 3.63(2.01-6.57) | 3.63(20.94) | 3.63(2.21) | 1.86(0.19) |
|  | Blood Albumin Decreased | 7 | 3.53(1.68-7.42) | 3.53(12.68) | 3.53(1.90) | 1.82(0.15) |
|  | Red Blood Cell Count Abnormal | 3 | 5.23(1.68-16.25) | 5.23(10.23) | 5.22( 2.02 ) | 2.38(0.71) |
|  | Blood Electrolytes Decreased | 3 | 6.17(1.99-19.18) | 6.17(12.95) | 6.15(2.38) | 2.62(0.95) |
|  | Blood Bilirubin Abnormal | 3 | 7.26(2.33-22.56) | 7.26(16.11) | 7.23(2.80) | 2.85(1.18) |
| Metabolism and Nutrition Disorders | Decreased Appetite | 491 | 6.12(5.60-6.70) | 6.01(2048.66) | 5.99( 5.55) | 2.58(0.92) |
|  | Dehydration | 262 | 6.64(5.87-7.5) | 6.57(1233.48) | 6.54(5.91) | 2.71(1.04) |
|  | Feeding Disorder | 30 | 3.81(2.66-5.46) | 3.81(62.04) | 3.8(2.82) | 1.93(0.26) |
|  | Failure To Thrive | 10 | 7.73(4.15-14.4) | 7.73( 58.32 ) | 7.7(4.58) | 2.94(1.28) |
|  | Cachexia | 9 | 5.53(2.87-10.65) | 5.53(33.29) | 5.51(3.19) | 2.46(0.80) |
| Musculoskeletal And Connective Tissue Disorders | Pathological Fracture | 5 | 3.34(1.39-8.03) | 3.34(8.17) | 3.33(1.60) | 1.74(0.07) |
| Nervous System Disorders | Dysgeusia | 82 | 3.48(2.80-4.32) | 3.47(143.77) | 3.46( 2.89) | 1.79(0.13) |
| Product Issues | Product Distribution Issue | 19 | 7.55(4.81-11.84) | 7.54(107.28) | 7.51(5.15) | 2.91(1.24) |
|  | Product Packaging Quantity Issue | 15 | 3.89(2.34-6.46) | 3.89(32.11) | 3.88(2.54) | 1.96(0.29) |
| Renal and Urinary Disorders | Hydronephrosis | 20 | 8.76(5.64-13.59) | 8.75(136.58) | 8.71(6.03) | 3.12(1.46) |
|  | Ureteric Obstruction | 11 | 28.69(15.8-52.09) | 28.68(288.69) | 28.19(17.12) | 4.82(3.15) |
|  | Urinary Bladder Haemorrhage | 3 | 4.26(1.37-13.24) | 4.26(7.47) | 4.25(1.65) | 2.09(0.42) |
| Reproductive System and Breast Disorders | Female Genital Tract Fistula | 3 | 4.96(1.6-15.42) | 4.96(9.46) | 4.95(1.92) | 2.31(0.64) |
| Surgical and Medical Procedures | Transfusion | 88 | 21.96(17.79-27.11) | 21.87(1729.63) | 21.59(18.10) | 4.43(2.77) |
|  | Stent Placement | 16 | 7.04(4.31-11.5) | 7.03(82.46) | 7.01(4.65) | 2.81(1.14) |
|  | Radiotherapy | 12 | 21.73(12.29-38.42) | 21.72(234.07) | 21.45( 13.31) | 4.42(2.75) |
|  | Paracentesis | 7 | 28.71(13.59-60.62) | 28.7(183.84) | 28.21(15.09) | 4.82(3.15) |
|  | Liver Operation | 6 | 70.07(30.94-158.69) | 70.05(391.36) | 67.17(33.89) | 6.07(4.39) |
|  | Colostomy | 6 | 9.30(4.17-20.74) | 9.29(44.16) | 9.25(4.72) | 3.21(1.54) |
|  | Infusion | 5 | 28.67(11.84-69.43) | 28.67(131.18) | 28.18(13.45) | 4.82(3.14) |
|  | Brain Operation | 4 | 5.16(1.93-13.76) | 5.16(13.36) | 5.14(2.26) | 2.36(0.69) |
|  | Liver Ablation | 4 | 143.23(51.51-398.3) | 143.21(518.75) | 131.6(55.93) | 7.04(5.32) |
|  | Hernia Repair | 3 | 3.69(1.19-11.45) | 3.69(5.86) | 3.68(1.43) | 1.88(0.21) |
|  | Nephrostomy | 3 | 24.41(7.81-76.35) | 24.41(66.35) | 24.06(9.27) | 4.59(2.91) |
|  | Fluid Replacement | 3 | 22.80(7.29-71.27) | 22.8(61.65) | 22.49(8.67) | 4.49(2.81) |
|  | Tumour Excision | 3 | 11.29(3.63-35.16) | 11.29(27.95) | 11.22(4.34) | 3.49(1.82) |
| Vascular Disorders | Jugular Vein Thrombosis | 3 | 6.07(1.95-18.85) | 6.06(12.64) | 6.05(2.34) | 2.60(0.93) |

ROR reporting odds ratio, CI confidence interval, PRR proportional reporting ratio, χ2 chi-squared, IC information

component, IC 025 the lower limit of 95% CI of the IC, EBGM empirical Bayesian geometric mean, EBGM 05

the lower limit of 95% CI of EBGM. *Emerging findings of Trifluridine/Tipiracil associated AEs from FAERS database
