# Supplementary material for: Signal mining and analysis of trifluridine/tipiracil adverse events based on real-world data from the FAERS database
Source: Front Pharmacol. 2024 Jul 23;15:1399998. doi: 10.3389/fphar.2024.1399998 (PMC11301057; doi:10.3389/fphar.2024.1399998)
Supplement: Supplementary file 6 [file Table4.docx]

| \| **Supplementary Table 4.** Signal strength of reports of Trifluridine/Tipiracil at the Preferred Terms (PTs) level in FAERS database（<65 years old） \| \| --- \| | | | | |
| --- | --- | --- | --- | --- | --- |
| **PT** | **N** | **ROR** | **(95%Cl) Lower** | **(95%Cl) Upper** |
| Death | 1182 | 26.28 | 24.69 | 27.98 |
| Disease Progression | 596 | 51.82 | 47.6 | 56.4 |
| Nausea | 352 | 3.48 | 3.12 | 3.87 |
| Fatigue | 290 | 2.86 | 2.54 | 3.21 |
| Vomiting | 204 | 3.59 | 3.12 | 4.12 |
| Diarrhoea | 190 | 2.54 | 2.2 | 2.93 |
| Decreased Appetite | 146 | 6.15 | 5.22 | 7.25 |
| Asthenia | 103 | 2.60 | 2.14 | 3.16 |
| Abdominal Pain | 102 | 3.15 | 2.59 | 3.83 |
| White Blood Cell Count Decreased | 87 | 6.15 | 4.98 | 7.60 |
| Inappropriate Schedule Of Product Administration | 84 | 2.48 | 2.00 | 3.07 |
| Dehydration | 82 | 6.96 | 5.59 | 8.65 |
| Constipation | 72 | 3.57 | 2.82 | 4.50 |
| Anaemia | 72 | 4.14 | 3.28 | 5.23 |
| Weight Decreased | 67 | 2.26 | 1.77 | 2.87 |
| Neutropenia | 65 | 3.21 | 2.51 | 4.10 |
| Pyrexia | 65 | 1.40 | 1.10 | 1.79 |
| Abdominal Pain Upper | 49 | 1.91 | 1.44 | 2.52 |
| Platelet Count Decreased | 42 | 4.06 | 3.00 | 5.50 |
| Intestinal Obstruction | 42 | 9.48 | 7.00 | 12.85 |
