# Supplementary material for: Signal mining and analysis of trifluridine/tipiracil adverse events based on real-world data from the FAERS database
Source: Front Pharmacol. 2024 Jul 23;15:1399998. doi: 10.3389/fphar.2024.1399998 (PMC11301057; doi:10.3389/fphar.2024.1399998)
Supplement: Supplementary file 7 [file Table6.docx]

| **Supplementary Table 6.** Signal strength of reports of Trifluridine/Tipiracil at the Preferred Terms (PTs) level in FAERS database（male） | | | | | | |
| --- | --- | --- | --- | --- | --- | --- |
| **PT** | **N** | **ROR** | **(95%Cl) lower** | | **(95%Cl) Upper** | |
| Death | 1651 | 8.35 | | 7.92 | | 8.8 |
| Disease Progression | 855 | 38.65 | | 36.01 | | 41.49 |
| Fatigue | 545 | 3.88 | | 3.56 | | 4.23 |
| Nausea | 435 | 4.28 | | 3.89 | | 4.71 |
| Diarrhoea | 392 | 3.21 | | 2.9 | | 3.55 |
| Decreased Appetite | 273 | 5.5 | | 4.88 | | 6.21 |
| Vomiting | 213 | 3.27 | | 2.86 | | 3.75 |
| White Blood Cell Count Decreased | 201 | 10.69 | | 9.3 | | 12.3 |
| Asthenia | 198 | 2.74 | | 2.38 | | 3.16 |
| Anaemia | 146 | 4.15 | | 3.53 | | 4.89 |
| Abdominal Pain | 135 | 3.58 | | 3.02 | | 4.24 |
| Dehydration | 132 | 5.53 | | 4.65 | | 6.56 |
| Constipation | 129 | 3.25 | | 2.73 | | 3.86 |
| Inappropriate Schedule Of Product Administration | 122 | 2.29 | | 1.92 | | 2.74 |
| Neutropenia | 109 | 3.9 | | 3.23 | | 4.72 |
| Malaise | 107 | 1.47 | | 1.21 | | 1.78 |
| Weight Decreased | 106 | 1.74 | | 1.44 | | 2.11 |
| Pyrexia | 88 | 1.24 | | 1.00 | | 1.53 |
| Platelet Count Decreased | 81 | 3.43 | | 2.76 | | 4.27 |
| Abdominal Pain Upper | 81 | 2.63 | | 2.11 | | 3.27 |

ROR: reporting odds ratio, CI: confidence interval.
