# Supplementary material for: Signal mining and analysis of trifluridine/tipiracil adverse events based on real-world data from the FAERS database
Source: Front Pharmacol. 2024 Jul 23;15:1399998. doi: 10.3389/fphar.2024.1399998 (PMC11301057; doi:10.3389/fphar.2024.1399998)
Supplement: Supplementary file 8 [file Image1.pdf]

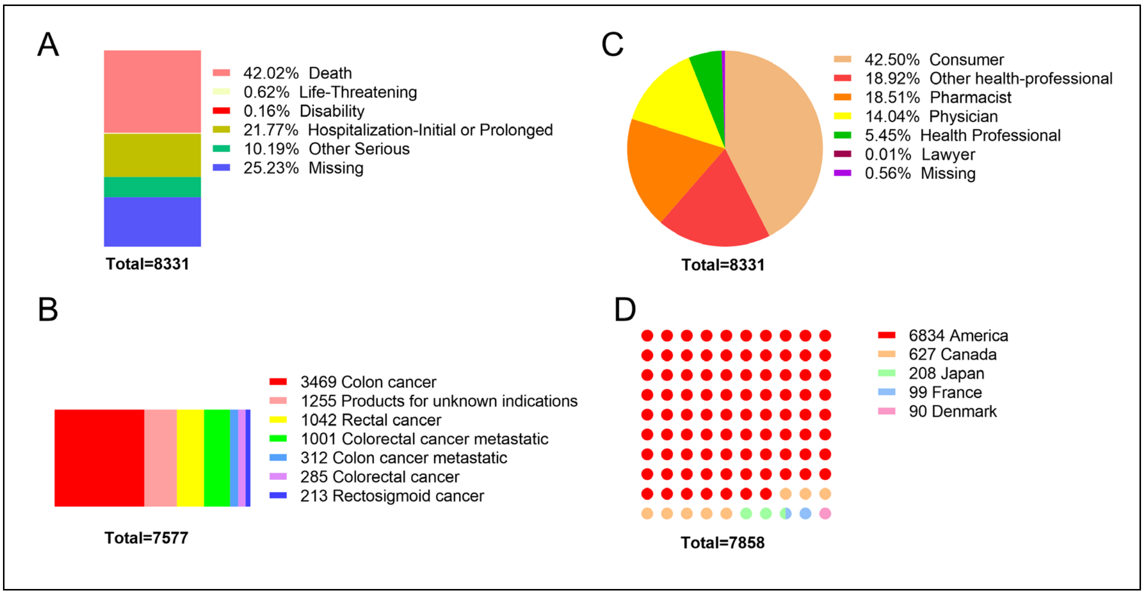

Supplementary Figure 1. Clinical characteristics of FTD/TPI-associated AEs A.Outcome in clinical characteristics. B. Indications in clinical characteristics (TOP seven). C. Reported Person in clinical characteristics. D. Reported Countries in clinical characteristics(Top five).
